# Sensitivity of midturbinate versus nasopharyngeal swabs for the detection of severe acute respiratory syndrome coronavirus 2 (SARS-CoV-2)

**DOI:** 10.1017/ice.2020.1326

**Published:** 2020-11-18

**Authors:** Alainna J. Jamal, Mohammad Mozafarihashjin, Eric Coomes, Sofia Anceva-Sami, Shiva Barati, Gloria Crowl, Amna Faheem, Lubna Farooqi, Christopher E. Kandel, Saman Khan, Angel X. Li, Henna Mistry, Aimee Paterson, Simon Plenderleith, Karren Prost, Susan Poutanen, Jeff Powis, Renée Schryer, Maureen Taylor, Lily Yip, Xi Zoe Zhong, Allison J. McGeer, Samira Mubareka

**Affiliations:** 1 Institute of Health Policy, Management and Evaluation, University of Toronto, Toronto, Canada; 2 Sinai Health System, Toronto, Canada; 3 Faculty of Medicine, University of Toronto, Toronto, Canada; 4 Sunnybrook Health Sciences Centre, Toronto, Canada; 5 Michael Garron Hospital, Toronto, Canada

## Abstract

To compare sensitivity of specimens for COVID-19 diagnosis, we tested 151 nasopharyngeal/midturbinate swab pairs from 117 COVID-19 inpatients using reverse-transcriptase polymerase chain reaction (RT-PCR). Sensitivity was 94% for nasopharyngeal and 75% for midturbinate swabs (*P* = .0001). In 88 nasopharyngeal/midturbinate pairs with matched saliva, sensitivity was 86% for nasopharyngeal swabs and 88% for combined midturbinate swabs/saliva.

Nasopharyngeal (NP) swabs are currently the preferred specimen collection method for coronavirus disease 2019 (COVID-19) diagnosis. However, there is increasing interest in midturbinate (MT) swabs because they cause less discomfort to patients, may be safer for healthcare workers to collect, and can be self-collected by adolescent and adult patients.^1,2^

Two small studies have compared the sensitivity of NP and MT swabs for detecting severe acute respiratory coronavirus virus 2 (SARS-CoV-2) and found comparable sensitivity early in illness.^2,3^ In our prospective cohort of inpatients with COVID-19 in Ontario, Canada, we compared the sensitivities of NP and saliva versus MT swabs.

## Methods

### Study population

The Toronto Invasive Bacterial Diseases Network (TIBDN) performs population-based surveillance for infectious diseases in Toronto and Peel (Ontario, Canada). The TIBDN clinical microbiology laboratories report specimens yielding SARS-CoV-2 to our study office. In this study, we enrolled consecutive inpatients with COVID-19 at 4 TIBDN hospitals from March 23 through May 22, 2020. The research ethics boards of all TIBDN hospitals approved this study.

### Data collection

Demographic and clinical data were collected from charts and patient interviews. Study staff collected NP and MT swabs and saliva from consenting patients at enrollment and 6 days later if the patient was still hospitalized. Swabs were obtained using standard procedures^4,5^ and placed in universal transport medium (UTM, COPAN Diagnostics, Murrietta, CA). Patients were asked to spit 1 teaspoon of saliva into a sterile container, then 2.5 mL phosphate-buffered saline was added. From April 14 through May 4, in a pilot test of alternative collection methods, saliva was provided by gargle and/or salivette; these specimens were excluded.

### Laboratory methods

Specimens were aliquoted and frozen at −80°C on the day of collection until processing at the Sunnybrook Health Sciences research microbiology laboratory (Toronto, Canada). RNA extractions were performed using the QIAamp Viral RNA Mini Kit (Qiagen, Venlo, The Netherlands). Reverse transcriptase polymerase chain reaction (RT-PCR) using the Luna Universal Probe One-Step RT-qPCR Kit (New England BioLabs, Ipswich, MA) was performed to detect the 5’ untranslated region and envelope (E) gene of SARS-CoV-2, with RNaseP as internal control. Cycle thresholds (Cts) were determined using Rotor-Gene Q software (Qiagen, Venlo, The Netherlands); results where all targets had Cts <40 were reported as positive.

### Statistical methods

Sensitivities of specimen types were calculated using as the denominator samples with any 1 specimen in the pair or triplet testing positive for SARS-CoV-2. The McNemar test was used to assess differences in sensitivity between specimen types. The Wilcoxon rank-sum test was used to compare the Cts of NP swabs when the MT tested positive versus negative. Two-sided *P* values <.05 were considered statistically significant. Analyses were performed using SAS version 9.4M6 software (SAS Institute, Cary, NC).

## Results

The 117 included inpatients were confirmed to have COVID-19 with a nasal, MT, or NP swab at or prior to admission. At admission, 88 of these patients (75%) had fever and 87 (74%) had cough. Their median age was 65 years (range, 23–106); 51 (44%) were female; 91 (78%) had ≥1 comorbidity; 14 (12%) were immunocompromised; 47 (40%) required intensive care; and 18 (15%) died. The median time from illness onset to specimen collection was 11 days (interquartile range [IQR], 8–16 days).

### NP and MT swab pairs

Overall, 151 NP/MT swab pairs were collected from these 117 patients, with at least 1 swab positive in 122 pairs (Table 1). Both NP and MT swabs were positive in 84 patients (69%), only the NP swab was positive in 31 patients (25%), and only the MT swab was positive in 7 patients (6%). The overall sensitivities of NP and MT swabs were 115 of 122 (94%; 95% CI, 90%–98%) and 91 of 122 (75%; 95% CI, 67%–83%), respectively (*P* = .0001, Table 1).

**Table 1.** Results of Testing of 151 NP and MT Swab Pairs for SARS-CoV-2 Among Hospitalized Patients With COVID-19, by Time From Illness Onset to Collection of Swab Pair

Time From Illness Onset to Collection of Swab Pair	No. (%) Swab Pairs			
	MT and NP Swabs Positive	Only NP Swab Positive	Only MT Swab Positive	MT and NP Swabs Negative
Day 0–7 (n=35)	28 (80)	5 (14)	0	2 (6)
Day 8+ (n=116)	56 (48)	26 (22)	7 (6)	27 (23)
All (n=151)	84 (56)	31 (21)	7 (5)	29 (19)

Note. COVID-19, coronavirus disease 2019; SARS-CoV-2, severe acute respiratory syndrome virus 2; MT, midturbinate; NP, nasopharyngeal.

The difference in sensitivity between NP and MT swabs increased with time from illness onset to specimen collection, but it was statistically significant even in the first week of illness: 33 of 33 (100%; 95% CI, 90%–100%) versus 28 of 33 (85%; 95% CI, 67%–94%), respectively (*P* = .03) (Table 1).

The median E gene Ct of NP swabs was 25 (IQR, 22–29) for positive (n = 84) and 32 (IQR, 29–35) for negative (n = 31) MT swabs (*P* < .0001). The E gene Cts of NP and MT swabs increased with time from illness onset (Spearman’s *ρ* = 0.4 [*P* < .0001] and *ρ* = 0.5 [*P* < .0001], respectively).

### NP swab, MT swab, and saliva triplets

A corresponding saliva specimen was available for 88 of the 151 NP/MT swab pairs. These 88 triplets were from 75 patients; at least 1 specimen was positive in 74 triplets. NP swabs detected SARS-CoV-2 in 64 of the 74 (86%; 95% CI 77-92%) triplets with at least one positive specimen, saliva detected 55 (74%; 95% CI 63-83%), MT swabs detected 49 (66%; 95% CI 54-77%), and MT and saliva in combination detected 65 (88%; 95% CI 78-94%) (Table 2).

**Table 2.** Results of Testing of 88 NP Swab/MT Swab/Saliva Triplets for SARS-CoV-2 Among Hospitalized Patients With COVID-19, by Time From Illness Onset to Collection of Triplet

Time From Illness Onset to Collection of Triplet	No. (%) Triplets							
	NP, MT, and Saliva Positive	Only NP and MT Positive	Only NP and Saliva Positive	Only MT and Saliva Positive	Only NP Positive	Only MT Positive	Only Saliva Positive	NP, MT, and Saliva Negative
Day 0–7 (n=19)	14 (74)	1 (5)	2 (11)	0	1 (5)	0	0	1 (5)
Day 8+ (n=69)	24 (35)	8 (12)	6 (9)	1 (1)	8 (12)	1 (1)	8 (12)	13 (19)
All (n=88)	38 (43)	9 (10)	8 (9)	1 (1)	9 (10)	1 (1)	8 (9)	14 (16)

Note. COVID-19, coronavirus disease 2019; SARS-CoV-2, severe acute respiratory syndrome virus 2; MT, mid-turbinate; NP, nasopharyngeal.

For all analyses, results were similar when only the first or last pair or triplet for each patient was analyzed (Supplementary Material online).

## Discussion

In this prospective cohort study of hospitalized patients with COVID-19, NP swabs were significantly more sensitive for SARS-CoV-2 detection than MT swabs. The difference in sensitivity between NP and MT swabs was greater later in illness. Saliva demonstrated intermediate sensitivity between MT and NP swabs.

We are aware of 2 studies comparing sensitivity of MT and NP swabs for SARS-CoV-2 detection.^2,3^ Pinninti et al,^3^ in inpatients similar to ours, reported NP swabs as more sensitive than MT swabs (80% vs 64% overall), with the difference in sensitivity increasing with time from illness onset. However, our difference in sensitivity between NP and MT swabs was statistically significant even in the first week of illness, whereas that of Pinninti et al was not. In an outpatient setting, Tu et al^2^ found that NP swabs detected all 52 patients with either swab positive, while MT swabs detected 50.

Our previous analysis comparing saliva to NP swabs in this cohort using a different testing platform indicated that saliva was less sensitive than NP swabs, particularly later in illness.^6^ The difference was not statistically significant in this sample, in which specimens were collected less frequently but over a longer period, likely due to sampling variability. More importantly, however, other studies have not found differences in sensitivities between NP swabs and saliva, particularly early in illness.^7,8^ Saliva is an appealing alternative to NP swabs, especially during swab shortages or in settings requiring repeated COVID-19 screening.

The fact that MT swabs and saliva in combination have a similar sensitivity to NP swabs may simply be because a second specimen adds sensitivity; repeated testing is known to improve sensitivity among patients with COVID-19.^9^ These data reinforce the concept that 1 specimen is not 100% sensitive and that patients with a high index of clinical suspicion for COVID-19 who test negative require repeated testing.

This study had several limitations. It included only hospitalized patients, with first study specimen at 1–27 days after diagnosis. Differences in sensitivity may be smaller at presentation. We only tested specimens on a single platform; results may vary between platforms, especially when Ct values are near the limit of detection. We did not perform sequencing or other validation of results in samples in which only 1 specimen yielded positive results, so some results may have been false positives.

In conclusion, this study of hospitalized patients found NP swabs to be 15% more sensitive than MT swabs for SARS-CoV-2 detection in the first week of COVID-19. Larger studies are needed, as are studies of specimens obtained for initial diagnosis and in milder illness. In the interim, we believe that the use of MT swabs for COVID-19 diagnosis should be approached with caution.

## Supplementary material

For supplementary material accompanying this paper visit https://doi.org/10.1017/ice.2020.1326. click here to view supplementary material
