# Supplementary material for: Sensitivity of midturbinate versus nasopharyngeal swabs for the detection of severe acute respiratory syndrome coronavirus 2 (SARS-CoV-2)
Source: Infect Control Hosp Epidemiol. 2020 Nov 18:1–3. doi: 10.1017/ice.2020.1326 (PMC7750656; doi:10.1017/ice.2020.1326)
Supplement: Supplementary file 1 [file S0899823X20013264sup001.docx]

**Supplementary Material**

**Table S1. Results of testing of NP and MT swab pairs for SARS-CoV-2 among hospitalized patients with COVID-19, by time from illness onset to collection of first swab pair per patient (n=117).**

|  | No. (%) swab pairs | | | |
| --- | --- | --- | --- | --- |
| Time from illness onset to collection of swab pair | MT and NP swabs positive | Only NP swab positive | Only MT swab positive | MT and NP swabs negative |
| Day 0-7 (n=34) | 27 (79) | 5 (15) | 0 | 2 (6) |
| Day 8+ (n=83) | 41 (49) | 17 (20) | 6 (7) | 19 (23) |
| All (n=117) | 68 (58) | 22 (19) | 6 (5) | 21 (18) |

Abbreviations: COVID-19=coronavirus disease 2019, SARS-CoV-2=severe acute respiratory syndrome virus 2, MT=mid-turbinate, NP=nasopharyngeal.

**Table S2. Results of testing of NP and MT swab pairs for SARS-CoV-2 among hospitalized patients with COVID-19, by time from illness onset to collection of last swab pair per patient (n=117).**

|  | No. (%) swab pairs | | | |
| --- | --- | --- | --- | --- |
| Time from illness onset to collection of swab pair | MT and NP swabs positive | Only NP swab positive | Only MT swab positive | MT and NP swabs negative |
| Day 0-7 (n=29) | 23 (79) | 4 (14) | 0 | 2 (7) |
| Day 8+ (n=88) | 42 (48) | 16 (18) | 7 (8) | 23 (26) |
| All (n=117) | 65 (56) | 20 (17) | 7 (6) | 25 (21) |

Abbreviations: COVID-19=coronavirus disease 2019, SARS-CoV-2=severe acute respiratory syndrome virus 2, MT=mid-turbinate, NP=nasopharyngeal.

**Table S3. Results of testing of NP swab/MT swab/saliva triplets for SARS-CoV-2 among hospitalized patients with COVID-19, by time from illness onset to collection of first triplet per patient (n=75).**

|  | No. (%) triplets | | | | | | | |
| --- | --- | --- | --- | --- | --- | --- | --- | --- |
| Time from illness onset to collection of triplet | NP, MT, and saliva positive | Only NP and MT positive | Only NP and saliva positive | Only MT and saliva positive | Only NP  positive | Only MT  positive | Only saliva positive | NP, MT, and saliva negative |
| Day 0-7 (n=19) | 14 (74) | 1 (5) | 2 (11) | 0 | 1 (5) | 0 | 0 | 1 (5) |
| Day 8+ (n=56) | 21 (38) | 6 (11) | 5 (9) | 1 (2) | 6 (11) | 1 (2) | 8 (14) | 8 (14) |
| All (n=75) | 35 (47) | 7 (9) | 7 (9) | 1 (1) | 7 (9) | 1 (1) | 8 (11) | 9 (12) |

Abbreviations: COVID-19=coronavirus disease 2019, SARS-CoV-2=severe acute respiratory syndrome virus 2, MT=mid-turbinate, NP=nasopharyngeal.

**Table S4. Results of testing of NP swab/MT swab/saliva triplets for SARS-CoV-2 among hospitalized patients with COVID-19, by time from illness onset to collection of last triplet per patient (n=75).**

|  | No. (%) triplets | | | | | | | |
| --- | --- | --- | --- | --- | --- | --- | --- | --- |
| Time from illness onset to collection of triplet | NP, MT, and saliva positive | Only NP and MT positive | Only NP and saliva positive | Only MT and saliva positive | Only NP  positive | Only MT  positive | Only saliva positive | NP, MT, and saliva negative |
| Day 0-7 (n=18) | 14 (78) | 1 (6) | 1 (6) | 0 | 1 (6) | 0 | 0 | 1 (6) |
| Day 8+ (n=57) | 20 (35) | 6 (11) | 3 (5) | 1 (2) | 7 (12) | 1 (2) | 8 (14) | 11 (19) |
| All (n=75) | 34 (45) | 7 (9) | 4 (5) | 1 (1) | 8 (11) | 1 (1) | 8 (11) | 12 (16) |

Abbreviations: COVID-19=coronavirus disease 2019, SARS-CoV-2=severe acute respiratory syndrome virus 2, MT=mid-turbinate, NP=nasopharyngeal.
